# Herpes Zoster as a Predictor of HIV Infection in Taiwan: A Population-Based Study

**DOI:** 10.1371/journal.pone.0142254

**Published:** 2015-11-04

**Authors:** Yuan-Ti Lee, Oswald Ndi Nfor, Disline Manli Tantoh, Jing-Yang Huang, Wen-Yuan Ku, Shu-Yi Hsu, Pei-Chieh Ko, Hung-Chang Hung, Cheng-Feng Jan, Yung-Po Liaw

**Affiliations:** 1 Division of Infectious Diseases, Department of Internal Medicine, Chung Shan Medical University Hospital, Taichung, Taiwan, ROC; 2 School of Medicine, Chung Shan Medical University, Taichung, Taiwan, ROC; 3 Institute of Medicine and School of Medicine, Chung Shan Medical University, Taichung, Taiwan, ROC; 4 Department of Public Health and Institute of Public Health, Chung Shan Medical University, Taichung City 40201, Taiwan; 5 Division of Gastroenterology, Department of Internal Medicine, Nantou Hospital, Ministry of Health and Welfare, Nantou, Taiwan, ROC; 6 Office of Physical Education, Chung Yuan Christian University, 200, Chung Pei Rd, Chung Li, Taiwan; 7 Department of Family and Community Medicine, Chung Shan Medical University Hospital, Taichung City 40201, Taiwan; Rega Institute for Medical Research, BELGIUM

## Abstract

This study aimed to investigate the association between herpes zoster (HZ) and human immunodeficiency virus (HIV). Data were retrieved from the Longitudinal Health Insurance Databases (LHID 2005 and 2010), Taiwan. The International Classification of Diseases, 9th Revision, Clinical Modification [ICD-9-CM] codes were used to identify Hz from 2001–2004. Identification of HIV infection was from 2005–2010. The hazard ratios of HIV among herpes zoster infected and non-herpes zoster infected patients were estimated using multiple Cox proportional hazard model. In general, 19685 participants were identified with Hz. The HIV incidence rates (per 10^4^ person-months) in herpes zoster infected and non-infected patients were 0.191(95% CI 0.130–0.280) and 0.079 (95% CI 0.074–0.084), respectively while the hazard ratio (HR) of HIV among infected individuals was 3.518 (95% CI 2.375–5.211). This study concludes that herpes zoster could be considered as a predictor of HIV infection especially among Asian populations, hence it is vital to test herpes zoster infected individuals for HIV antibodies.

## Introduction

Infection with varicella-zoster virus causes chicken pox. The virus is capable of remaining latent in the neuronal cell bodies after resolution of the initial occurrence of chicken pox. The immune system eliminates it from most locations and suppresses its reactivation but sometimes, suppression can fail. Herpes zoster (Hz) occurs as a result of reactivation of the virus. It is common in people with compromised immune systems due to aging and psychological stress and other infections such as HIV [1, 2]. Occurrence of Hz in young adults is uncommon. However, infection among young adults have been reported in sub-Saharan Africa where it has been regarded as one of the strongest predictors of human immunodeficiency virus type 1 [1–4]. The occurrence of Hz in HIV-infected individuals is 10 times more often than in non HIV-infected individuals [5, 6]. Nonetheless, as far as we know, there is limited available information on the association between Hz and HIV in Asia. The objective of study was to investigate the association between Hz and HIV in Taiwan.

## Materials and Methods

Data from the National Health Insurance Research Database released by the National Health Research Institute of Taiwan was used in this study. Information was retrieved from the LHID 2005 and 2010 each composing of 1000000 enrollees. The National Health Insurance program finances health care for 99% of all residents of Taiwan. This database contains comprehensive information such as demographic data, dates of clinical visits, diagnostic codes, enrollment dates, details of prescription and expenditure. Personal information on family history, lifestyle and habits (e.g., smoking, alcohol use) were not available in the database. The dataset consisted of de-identified secondary data. The ICD-9-CM codes used to identify HZ were 053–053.9. HIV diagnosis was from 2005–2010. Low-income individuals were defined as having monthly per capita income below the minimum living expenses standard for selected areas (according to the Ministry of Health and Welfare, Taiwan Social Assistance Act). The minimum living expense standard was defined as 60% of the average monthly disposable income for each region. The family property is not supposed to exceed a certain amount, as determined by the central or municipal authorities in the corresponding year. Individuals diagnosed with HIV from 2001–2004 were excluded from the study. All patients were followed until death, dropout from the Health Insurance program or diagnosis of HIV from 2005 until 2010.

The hazard ratios of HIV among herpes zoster infected and non-infected patients were estimated using multiple Cox proportional hazard model. Potential confounders included age, low-income households, geographical area and comorbidity such as syphilis (ICD-9:091–097), genital warts (ICD-9:078.1), genital ulcer disease (ICD-9:054.1), non-gonococcal urethritis (ICD-9:099.4), balanitis (ICD-9:607.1), gonorrhea (ICD-9:098), Pthirus pubis (ICD-9:132) as well as other sexually transmitted diseases (ICD-9:099) like Trichomonas vaginitis (ICD-9:131) and Chlamydia (ICD-9:078.8). Adjustments were also made for substance abuse (ICD-9:304,305), alcoholic psychoses (ICD-9:291) and drug psychoses (ICD-9:292). Discrete and continuous variables in herpes zoster infected and non-infected individuals were compared using the chi-square and t-tests.

## Results

**Fig 1:**
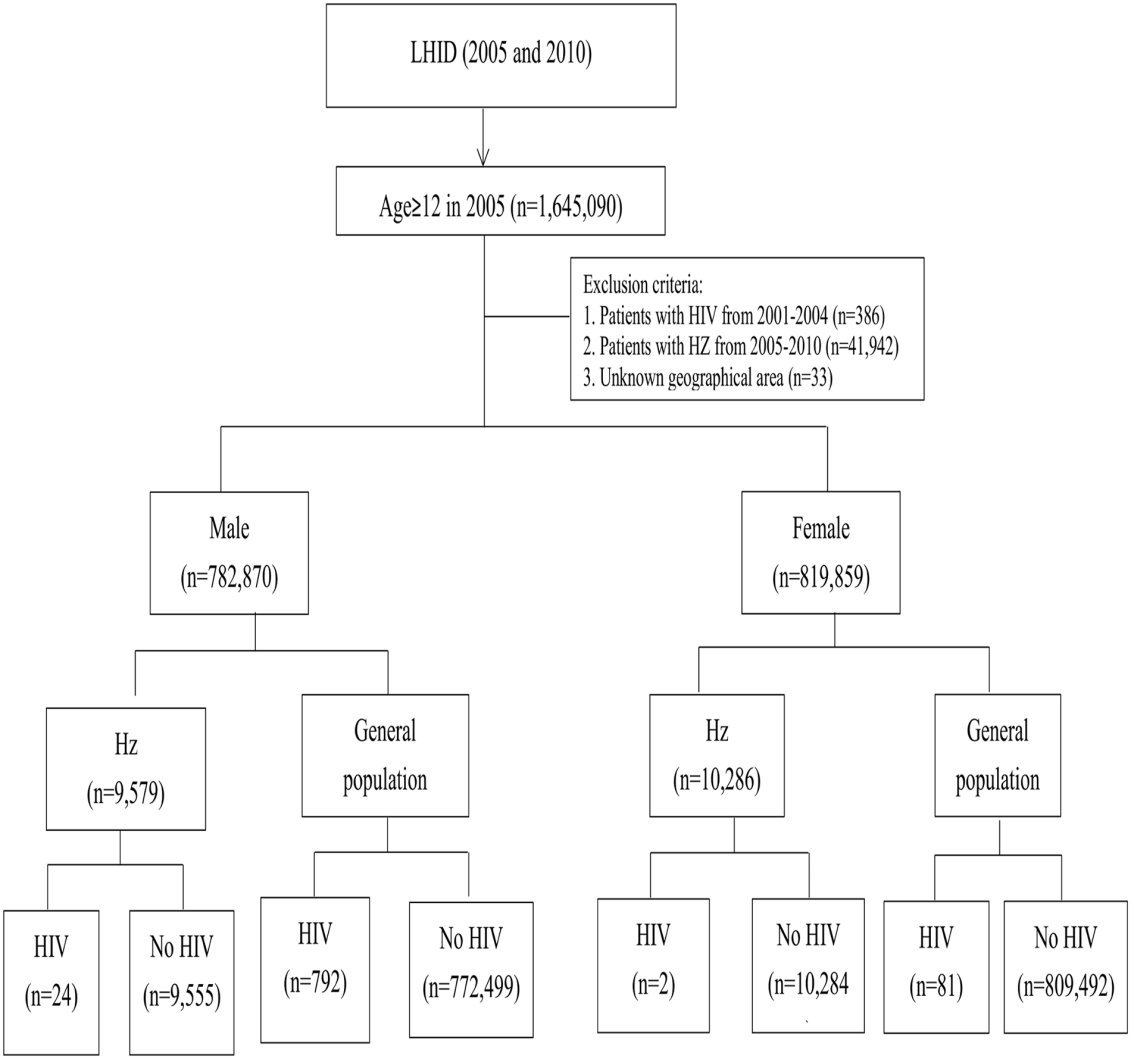
Flow chart of study participants.

**Fig 2:**
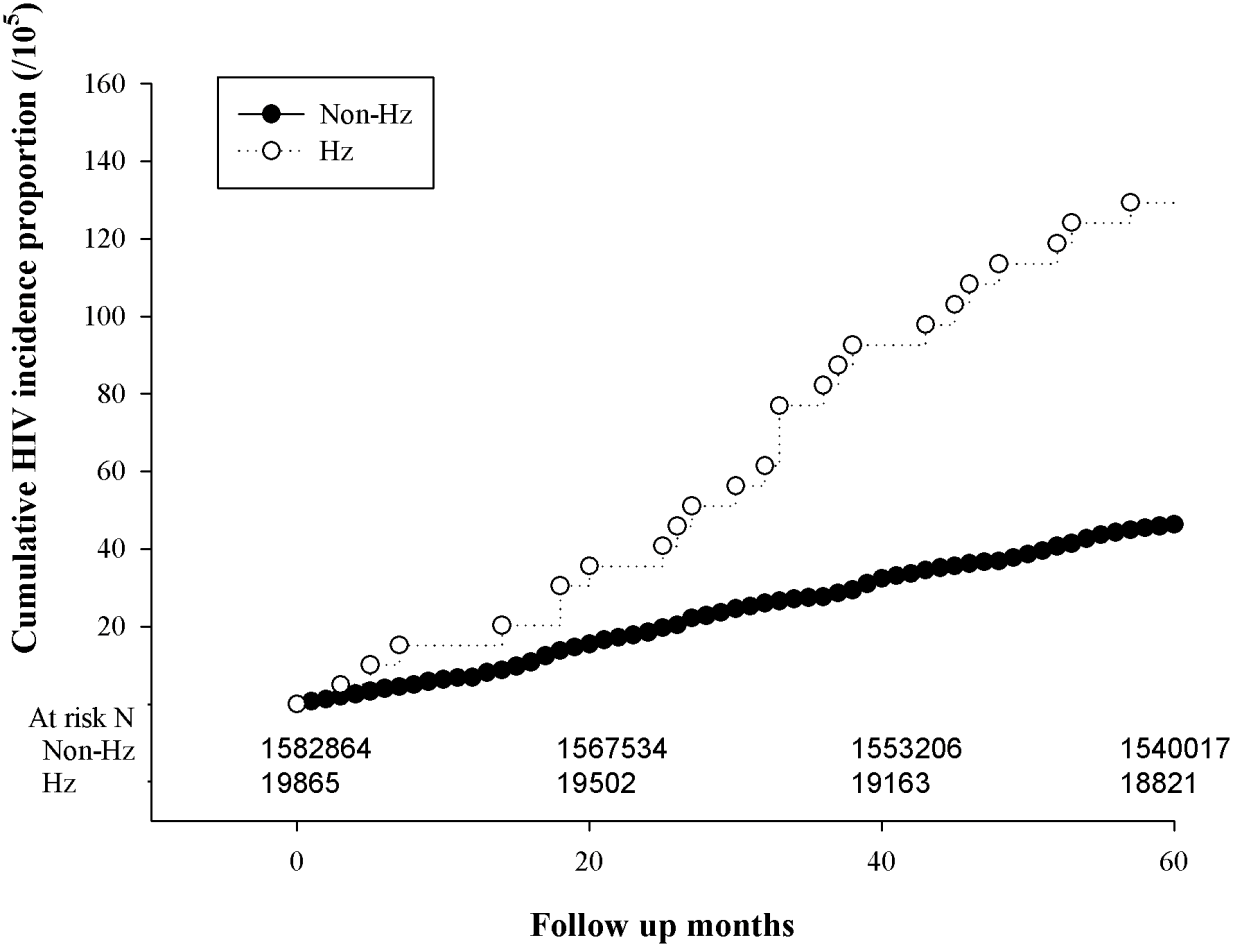
KM plots of cumulative HIV incidence proportion among HZ individuals.

A total of 19865 cohorts (9579 men and 10286 women) were diagnosed with Hz from 2001–2004 (Fig 1). The prevalence proportion (per 10^4^ persons) of HIV was 13.09 (95% CI 8.06–18.12) and 5.52 (95% CI 5.15–5.88) among herpes zoster infected and non-herpes zoster infected patients respectively (Table 1). The incidence of HIV (per 104 person-months) was 0.191 (95% CI 0.130–0.280) and 0.079 (95% CI 0.074–0.084) for both the infected and non-infected patients, respectively (Table 2). Kaplan-Meier analysis showed that the cumulative incidences of AIDS were 0.12 and 0.04% among the infected and non-infected patients (Fig 2).

**Table 1:** Demographic characteristics of patients with/without Hz.

Variable	Total		p-value
	Hz (N = 19,865)	Non- Hz (N = 1,582,864)	
**Age (y) in 2005**			
Mean (s. d.)	53.70 (18.69)	39.86 (17.58)	< .0001
**Hiv**			< .0001
Yes*	26 (0.13%)	873 (0.06%)	
No	19,839 (99.87%)	1,581,991 (99.94%)	
**Sex**			0.0759
Male	9,579 (48.22%)	773,291 (48.85%)	
Female	10,286 (51.78%)	809,573 (51.15%)	
**Low income**			0.2235
Yes	149 (0.75%)	13,119 (0.83%)	
**Geographical area**			< .0001
Taipei	7,202 (36.25%)	581,263 (36.72%)	
Northern	2,376 (11.96%)	222,833 (14.08%)	
Central	4,121 (20.75%)	286,451 (18.10%)	
Southern	2,808 (14.14%)	220,252 (13.91%)	
Kao-Ping	2,869 (14.44%)	236,448 (14.94%)	
Eastern	489 (2.46%)	35,617 (2.25%)	
**Comorbidity**			
Syphilis	76 (0.38%)	3,335 (0.21%)	< .0001
Gonorrhea	73 (0.37%)	3,859 (0.24%)	0.0005
Genital Warts	1,668 (8.40%)	91,186 (5.76%)	< .0001
Other sexually transmissible diseases	583 (2.93%)	35,315 (2.23%)	< .0001
Substance abuse	194 (0.98%)	15,882 (1.00%)	0.7066
Alcoholic psychoses	193 (0.97%)	16,340 (1.03%)	0.3997
Drug psychoses	43 (0.22%)	4,036 (0.25%)	0.2842

* The prevalence (/10^4^) of HIV was 5.52 (95% C.I. 5.15–5.88) in non-Hz and 13.09 (95% C.I. 8.06–18.12) in Hz infected.

**Table 2:** Incidence of HIV among patients with/without herpes zoster.

	Incidence rate (per 10^4^ person-months)			
	Cases/ person-month (*10^4^)	HIV cases	Estimate	95% C.I.
No herpes zoster	1104.27	873	0.079	0.074–0.084
Herpes zoster	13.63	26	0.191	0.130–0.280

The hazard ratio of HIV among herpes zoster infected patients was 3.518 (95% CI 2.375–5.211) as shown in Table 3. Among infected patients ≤ 50 the HR of HIV was 4.052 (95% CI 2.625–6.255) and 2.385 (95% CI 0.331–17.199) in men and women respectively (Table 4). Male and female patients above the age of 50 years had HRs of 4.688 (95% CI 1.405–15.638) and 4.916 (CI 0.595–40.631) as seen in Table 5. Compared with all groups the HRs were higher in the 30–40 (HR = 6.227, 95% C.I. 3.197–12.129) and 50–60 years of age range (HR = 6.787, 95% C.I. 2.026–22.737). For the interaction between HZ and age, the p value for interaction term (0.5960) was not significant. We tested for herpes zoster effect over time. The chi-square value was 0.0893 and the degree freedom was 1 while the associated p value was 0.7651, hence the proportional hazards was satisfied. The recurrence rate of HZ was higher in HIV infected (53.85%) than the non-HIV individuals (8.37%). The OR of HZ in HIV infected individuals was 22.24 (10.71–49.11) after adjustments were made for age, sex, low-income, area and co-morbidity.

**Table 3:** Adjusted Hazard ratio for HIV among patients with HZ.

Variable	HR	95% CI
**Hz** (ref: No)		
Yes	3.518	2.375–5.211
**Age** (per 1 year)	0.963	0.959–0.968
**Sex** (ref: Female)		
Male	9.389	7.486–11.776
**Low income** (ref: No)		
Yes	1.040	0.557–1.943
**Geographical area** (ref: Taipei)		
Northern	0.965	0.788–1.182
Central	0.897	0.740–1.086
Southern	0.873	0.703–1.086
Kao-Ping	1.139	0.941–1.377
Eastern	0.801	0.477–1.345
**Comorbidity** (ref: No)		
Syphilis	23.568	17.547–31.654
Gonorrhea	3.145	1.919–5.154
Genital Warts	1.320	1.046–1.665
Other sexually transmitted diseases	2.405	1.895–3.053
Substance abuse	23.069	15.492–34.351
Alcoholic psychoses	0.179	0.117–0.274
Drug psychoses	5.548	3.785–8.132

**Table 4:** Adjusted Hazard ratio of HIV among male and female patients (≤ 50) with HZ stratified by sex.

	Total		Male		Female	
Variable	HR	95% CI	HR	95% CI	HR	95% CI
**Hz** (ref: No)						
Yes	3.912	2.561–5.977	4.052	2.625–6.255	2.385	0.331–17.199
**Age** (per 1 year)	0.985	0.978–0.991	0.985	0.978–0.992	0.982	0.960–1.005
**Low income** (ref: No)						
Yes	1.063	0.55–2.054	1.199	0.62–2.318	-	-
**Geographical area** (ref: Taipei)						
Northern	0.997	0.813–1.224	0.928	0.746–1.154	1.906	1.014–3.581
Central	0.894	0.734–1.089	0.861	0.699–1.06	1.352	0.705–2.592
Southern	0.850	0.678–1.067	0.832	0.656–1.055	1.161	0.536–2.515
Kao-Ping	1.174	0.966–1.426	1.184	0.968–1.448	1.076	0.512–2.265
Eastern	0.869	0.518–1.46	0.875	0.511–1.496	0.783	0.106–5.801
**Comorbidity** (ref: No)						
Syphilis	23.96	17.762–32.321	25.428	18.717–34.545	3.313	0.436–25.156
Gonorrhea	2.981	1.813–4.899	2.972	1.803–4.900	-	-
Genital Warts	1.318	1.037–1.676	1.330	1.035–1.709	1.193	0.517–2.754
Other sexually transmitted diseases	2.291	1.796–2.924	2.232	1.732–2.877	2.406	0.952–6.083
Substance abuse	19.842	13.164–29.906	16.589	10.644–25.856	98.824	34.253–285.116
Alcoholic psychoses	0.187	0.121–0.289	0.199	0.125–0.318	0.198	0.061–0.647
Drug psychoses	5.397	3.667–7.944	5.515	3.670–8.287	5.915	1.810–19.332

**Table 5:** Adjusted Hazard ratio of HIV among male and female patients (≥ 50) with HZ stratified by sex.

	Total		Male		Female	
Variable	HR	95% CI	HR	95% CI	HR	95% CI
**Hz** (ref: No)						
Yes	4.714	1.658–13.405	4.688	1.405–15.638	4.916	0.595–40.631
**Age** (per 1 year)	0.921	0.880–0.964	0.910	0.862–0.960	0.9620	0.883–1.048
**Low income** (ref: No)						
Yes	2.734	0.358–20.862	2.905	0.376–22.415	-	-
**Geographical area** (ref: Taipei)						
Northern	0.202	0.027–1.533	0.342	0.043–2.703	-	-
Central	1.172	0.512–2.682	1.712	0.659–4.446	0.322	0.039–2.684
Southern	1.660	0.742–3.711	2.476	0.977–6.275	0.396	0.047–3.316
Kao-Ping	0.826	0.319–2.138	1.374	0.487–3.882	-	-
Eastern			-	-		
**Comorbidity** (ref: No)						
Syphilis	4.987	0.616–40.385	6.915	0.820–58.290	-	-
Gonorrhea	-	-	-	-	-	-
Genital Warts	2.361	0.914–6.097	2.449	0.846–7.089	2.087	0.254–17.114
Other sexually transmitted diseases	3.256	0.955–11.101	2.460	0.541–11.179	8.491	1.024–70.405
Substance abuse	89.26	25.545–311.893	94.639	26.523–337.69	-	-
Alcoholic psychoses	0.034	0.006–0.204	0.034	0.006–0.202	-	-
Drug psychoses	4.759	0.558–40.581	4.569	0.529–39.482	-	-

## Discussion

To our knowledge, this is the first study to investigate the association between Hz and HIV by gender and age in Taiwan. In this study, the hazard ratio of HIV among individuals with herpes zoster reached statistical significance. When calculated for 10-year age group, the HRs were found to be higher in the 30–40 and 50–60 age groups. It has been indicated that patients with HZ (≤ 50 years) should be screened for screened for HIV [7]. Other studies have shown that Hz is more common in adults ≥ 50 years [8, 9]. This may be attributed to decreased immunocompetence associated with aging. Most studies have described the incidence of Hz among HIV patients [5] and have also evaluated the impact of highly active antiretroviral therapy (HAART) on incidence of Hz in HIV patients [10]. However, few studies have described Hz as a predictor of HIV. In this study, HIV prevalence was significantly high among patients with Hz patients. This shows that people with HZ are more likely to have HIV; therefore, herpes zoster may be used as a predictor of HIV infection. How these two viral infections interact is still unclear. Contraction of HIV by Hz patients can be explained in terms of sexual activities of the participants as most men below 50 had a significant relationship with other risk factors for HIV. The presence of herpes zoster lesions may have facilitated the acquisition of HIV. In this study, the interaction between HZ and age was not significant. The recurrence rate of HZ was found to be higher in HIV infected than the non-HIV individuals. There were some limitations to this study. First, patients with mild Hz who did not visit the hospital may have probably been misclassified as not having Hz. This may have probably resulted in an under estimation of HR of HIV. Other cases (e.g., herpes simplex) might have been misdiagnosed as zoster as has been previously reported [11–13]. Second, we couldn’t compare the HR of HIV between those who received treatment for HZ and those who never received it. Making such comparisons would have been useful in understanding the benefits of herpes zoster treatment. Moreover, HIV incidence among participants without HZ, those with recurrent HZ and those without recurrent HZ were not determined. It is our hope that such comparison can be assessed in future research. Furthermore, interaction was not tested between HIV and the 13 co-morbid conditions. Some of the co-morbid conditions were considered under “other sexually transmitted diseases” during analysis.

## Conclusion

This study shows a clear association between HZ and HIV suggesting that herpes zoster could be considered as a predictor of HIV infection especially among Asian populations. It is vital to test infected individuals for HIV antibodies.

## References

[1] Tyndall MW, Nasio J, Agoki E, Malisa W, Ronald AR, Ndinya-Achola JO, et al Herpes zoster as the initial presentation of human immunodeficiency virus type 1 infection in Kenya. Clinical infectious diseases. 1995;21(4):1035–7. 8645797 10.1093/clinids/21.4.1035

[2] Colebunders R, Mann JM, Francis H, Hila K, Izaley L, Ilwaya M, et al Herpes zoster in African patients: a clinical predictor of human immunodeficiency virus infection. Journal of Infectious Diseases. 1988;157(2):314–8. 3335810 10.1093/infdis/157.2.314

[3] Lindan CP, Allen S, Serufilira A, Lifson AR, Van de Perre P, Chen-Rundle A, et al Predictors of mortality among HIV-infected women in Kigali, Rwanda. Annals of internal medicine. 1992;116(4):320–8. 1733389 10.7326/0003-4819-116-4-320

[4] Hira S, Ngandu N, Wadhawan D, Nkowne B, Baboo K, Macuacua R, et al Clinical and epidemiological features of HIV infection at a referral clinic in Zambia. JAIDS Journal of Acquired Immune Deficiency Syndromes. 1990;3(1):87–91. 2293647

[5] Buchbinder SP, Katz MH, Hessol NA, Liu JY, O'Malley PM, Underwood R, et al Herpes zoster and human immunodeficiency virus infection. Journal of Infectious Diseases. 1992;166(5):1153–6. 1308664 10.1093/infdis/166.5.1153

[6] Veenstra J, Krol A, van Praag RM, Frissen PJ, Schellekens PTA, Lange JM, et al Herpes zoster, immunological deterioration and disease progression in HIV-1 infection. Aids. 1995;9(10):1153–8. 8519451 10.1097/00002030-199510000-00006

[7] Gnann JW, Whitley RJ. Herpes Zoster. New England Journal of Medicine. 2002;347(5):340–6. doi: 10.1056/NEJMcp013211. 12151472 10.1056/NEJMcp013211

[8] Hope-Simpson RE. The nature of herpes zoster: a long-term study and a new hypothesis. Journal of the Royal Society of Medicine. 1965;58(1):9–20. 10.1177/003591576505800106 PMC1898279 14267505

[9] Ragozzino M, MELTON L III, Kurland L, Chu C, Perry H. Population-based study of herpes zoster and its sequelae. Medicine. 1982;61(5):310–6. 6981045 10.1097/00005792-198209000-00003

[10] Moanna A, Rimland D. Decreasing incidence of herpes zoster in the highly active antiretroviral therapy era. Clinical infectious diseases. 2013;57(1):122–5. doi: 10.1093/cid/cit165 23487391 10.1093/cid/cit165

[11] Helgason S, Sigurdsson JA, Gudmundsson S. The clinical course of herpes zoster: a prospective study in primary care. European Journal of General Practice. 1996;2(1):12–6.

[12] Rübben A, Baron J, GRUSSENDORF‐CONEN EI. Routine detection of herpes simplex virus and varicella zoster virus by polymerase chain reaction reveals that initial herpes zoster is frequently misdiagnosed as herpes simplex. British Journal of Dermatology. 1997;137(2):259–61. 9292077 10.1046/j.1365-2133.1997.18161913.x

[13] Kalman CM, Laskin OL. Herpes zoster and zosteriform herpes simplex virus infections in immunocompetent adults. The American journal of medicine. 1986;81(5) 10.1016/0002-9343(86)90343-8 3022586

